# Investigation of CD28 Gene Polymorphisms in Patients with Sporadic Breast Cancer in a Chinese Han Population in Northeast China

**DOI:** 10.1371/journal.pone.0048031

**Published:** 2012-10-25

**Authors:** Shuang Chen, Qing Zhang, Liming Shen, Yanhong Liu, Fengyan Xu, Dalin Li, Zhenkun Fu, Weiguang Yuan, Da Pang, Dianjun Li

**Affiliations:** 1 Department of Immunology, Harbin Medical University, Harbin, China; 2 Department of Tumor Cell Biology, Institute of Cancer Prevention and Treatment, Harbin Medical University, Harbin, China; 3 Department of Surgery, the Third Affiliated Hospital of Harbin Medical University, Harbin, China; 4 Department of Laboratory Medicine, the Second Hospital of Harbin Medical University, Harbin, China; University of North Carolina School of Medicine, United States of America

## Abstract

**Background:**

CD28 is one of a number of costimulatory molecules that play crucial roles in immune regulation and homeostasis. Accumulating evidence indicates that immune factors influence breast carcinogenesis. To clarify the relationships between polymorphisms in the CD28 gene and breast carcinogenesis, a case-control study was conducted in women from Heilongjiang Province in northeast of China.

**Methodology/Principal Findings:**

Our research subjects consisted of 565 female patients with sporadic breast cancer and 605 age- and sex-matched healthy controls. In total, 12 single nucleotide polymorphisms (SNPs) in the CD28 gene were successfully determined using the polymerase chain reaction-restriction fragment length polymorphism (PCR-RFLP) method. The relationship between the CD28 variants and clinical features, including histological grade, tumor size, lymph node metastasis, human epidermal growth factor receptor 2 (C-erbB2), estrogen receptor (ER), progesterone receptor (PR), and tumor protein 53 (P53) status were analyzed. A statistically significant association was observed between rs3116496 and breast cancer risk under different genetic models (additive P = 0.0164, dominant P = 0.0042). Different distributions of the rs3116496 ‘T’ allele were found in patients and controls, which remained significant after correcting the P value for multiple testing using Haploview with 10,000 permutations (corrected P = 0.0384). In addition, significant associations were observed between rs3116487/rs3116494 (D’ = 1, r^2^ = 0.99) and clinicopathological features such as C-erbB2 and ER status, in breast cancer patients.

**Conclusions/Significance:**

Our findings indicate that CD28 gene polymorphisms contribute to sporadic breast cancer risk and have a significant association with clinicopathological features in a northeast Chinese Han population.

## Introduction

Breast cancer is the most commonly diagnosed female cancer worldwide. It is now estimated as the leading cause of cancer death among women in developing countries [1]. The etiology of breast cancer has not been completely elucidated, but is thought to be multifactorial, with both environmental and genetic factors [2].

The immune system serves as an important natural barrier to cancer development. Innate and adaptive responses are carefully orchestrated through soluble and membrane-bound receptors to eliminate precancerous cells and control neoplastic progression. Avoiding immune destruction has been considered a hallmark of cancer [3], [4]. It is therefore of great interest to identify immune genes that influence susceptibility to breast cancer.

Costimulatory signals contribute to immune regulation and homeostasis [5], [6], [7], [8]. CD28, as the best characterized costimulatory molecule, is constitutively expressed by the vast majority of T lymphocytes. Upon interaction with its ligand B7-1 (CD80)/B7-2 (CD86), CD28 mediates signals that promote T lymphocyte differentiation and proliferation, and enhance antibody production of B lymphocytes [9], [10], [11]. T lymphocytes are the central regulator of the anti-tumor adaptive immune response [12]. Lack of costimulatory signals leads to T lymphocyte tolerance and anergy [6]. It has been demonstrated that deficiencies in both CD28 and inducible costimulator (ICOS) pathways result in complete T lymphocyte tolerance *in vitro* and *in vivo* [13], [14]. Both CD4+ and CD8+ memory T cells need CD28 costimulation to achieve maximal expansion and pathogen clearance [15]. The blockade of the CD28-B7 interaction has been used to down-regulate the activation of the immune system in autoimmune diseases [16]. Cytotoxic T-lymphocyte-associated antigen 4 (CTLA-4), which competes with CD28 for B7 binding, has been widely accepted as a new promising target for cancer immunotherapy. It has been reported that the anti-CTLA-4 monoclonal antibody ipilumumab blocks the activation of immunosuppressive CTLA-4, and thus induces tumor regression [17], [18].

SNPs are the most common genetic variations which influence interindividual predisposition to breast carcinogenesis and prognosis [19]. SNP association analysis has provided valuable information about the genetic susceptibility of breast cancer [20]. The human CD28 gene is located in the chromosome 2q33 region. Polymorphisms in the CD28 gene were previously shown to be genetically associated with autoimmune diseases, such as rheumatoid arthritis and Bechet’s disease [21], [22]. Several compelling reports characterized the association between this susceptibility loci and cervical cancer risk in different ethnic groups [23], [24], [25], [26], [27]. Chen et al. [23] and Ivansson et al. [24] found that the rs3116496 TT genotype was associated with a low risk of cervical cancer in a Chinese Han and a Swedish population, respectively. Conversely, Guzman et al. [26] observed that the rs3116496 TT genotype was associated with an increased cervical cancer risk when combined with the IFN+847AA genotype, in a Brazilian population. Pawlak et al. [25] demonstrated that rs3116496 was not correlated with cervical squamous cell carcinoma, when taking all evaluated patients into consideration.

No case-control study of CD28 gene polymorphisms in sporadic breast cancer has been reported. Given the association between polymorphisms in the CD28 gene region and cervical cancer risk in different populations, as well as the potential role of costimulatory molecules in carcinogenesis, we examined the association between CD28 polymorphisms and breast cancer risk and tumor pathology. Our data provides the first evidence for the involvement of the human CD28 gene in breast cancer.

## Results

### Polymorphism Detection

A total of 565 breast cancer patients (**Table 1**) and 605 healthy controls were involved in this case-control study. SNPs were excluded if they deviated from HWE (P<0.05), which did not occur in this study. 12 SNPs (rs3181097, rs3181100, rs1181388, rs10932017, rs4673259, rs3769684, rs3116487, rs3116494, rs3116496, rs12693993, rs3769686, and rs35593994) were successfully analyzed. Two SNPs (rs1879877 and rs2140148) genotyped were excluded, because more than 10% of the data was missing (resulting from the lack of sufficient genomic DNA in the samples).

**Table 1:** Clinicopathologic information of breast cancer patients.

Clinicopathologic information	Case, No. (%)
Tumor Type	
IDC^1^	471 (83.36)
ILC^2^	14 (2.48)
Intraductal carcinoma	41 (7.26)
Mucinous adenocarcinoma	15 (2.65)
Others	24 (4.25)
Tumor Size	
With the diameter less than 2 cm	187 (33.10)
With the diameter of 2 to 5 cm	252 (44.60)
With the diameter more than 5 cm	27 (4.78)
Unknown	99 (17.52)
LN^3^ involvement	
Positive	235 (41.59)
Negative	310 (54.87)
Unknown	20 (3.54)
ER^4^	
Positive	282 (49.91)
Negative	202 (35.75)
Unknown	81 (14.34)
PR^5^	
Positive	346 (61.24)
Negative	136 (24.07)
Unknown	83 (14.69)
P53^6^	
Positive	146 (25.84)
Negative	321 (56.81)
Unknown	98 (17.35)
C-erbB2^7^	
Positive	187 (33.10)
Negative	292 (51.68)
Unknown	86 (15.22)

1 IDC, infiltrative ductal carcinoma.

2 ILC, Infiltrative lobular carcinoma.

3 LN, lymph node.

4 ER, estrogen receptor.

5 PR, progesterone receptor.

6 P53, tumor protein 53.

7 C-erbB2, human epidermal growth factor receptor 2.

### Linkage Disequilibrium

All SNP detected were located in the chromosome 2q33 region. Linkage disequilibrium (LD) patterns were analyzed using our own material (data from 565 patients and 605 controls) with Haploview 4.1 software. LD was expressed by both D’ and r^2^. The strongest LD was observed between rs3116487 and rs3116494, with D’ = 1 and r^2^ = 0.99. The remaining LDs were presented in the LD plot (**Figure S1)**. Using the methods of Gabriel et al. [28] described in the statistical analysis section, which defines confidence limits for strong LD (upper, 0.85; lower, 0.70), and the confidence interval maximums for strong recombination (upper, 0.85) and for strong LD (upper, at least 0.8), two LD blocks were identified from the CD28 gene region and its flanking sequence. As shown in **Figure S2**, LD block 1 was composed of rs3181097, rs35593994, rs3181100, rs1181388, rs10932017, rs4673259, and rs3769684, while block 2 was composed of only rs3116487 and rs3116494. In LD block 1, the haplotype A_rs3181097_G_rs35593994_C_rs3181100_T_rs1181388_C_rs10932017_C_rs4673259_C_rs3769684_ was the most common haplotype (45.7% of cases and 48.3% of controls). Haplotye G_rs3181097_A_rs35593994_C_rs3181100_C_rs1181388_T_rs10932017_T_rs4673259_T_rs3769684_ was less common (21.9% of cases and 20.0% of controls). rs3116487 and rs3116494 (D’ = 1 r^2^ = 0.99) belonged to LD block 2, and they constructed only two haplotypes. The haplotype C_rs3116487_A_rs3116494_ was the most common haplotype (92.6% of cases and 91.6% of controls), while G_rs3116487_G_rs3116494_ was less frequently observed (7.4% of cases and 8.4% of controls).

### Frequencies of Gene Variants in Cases and Controls

The distributions of the CD28 genotypes are shown in **Table 2**. The odds ratios and 95% CIs in **Table 2** are presented for the dominant genetic model identified by a logistic regression analysis using Plink 1.07 software. At the single SNP level, a trend in genotype distribution was observed for rs3116496 (CC vs. CT vs. TT additive P = 0.0164, CC+CT vs. TT dominant P = 0.0042). Although there was also a trend for a higher proportion of rs35593994 genotype (AA+AG) in the dominant genetic model in patients with breast cancer compared with controls, the trend did not achieve statistical significance (P = 0.0989). Moreover, the rs3116487 GG and rs3116494 GG genotypes had a lower prevalence in breast cancer patients than in healthy controls, under the recessive genetic model. For SNP rs3119686, no GG genotype was detected in this study and the frequency of the ‘G’ allele is only 1.75%. Thus, only the allelic P value for rs3119686 was calculated between cases and controls in **Table 3**. As shown in **Table 3**, a higher prevalence of ‘C’ alleles was observed (P = 0.0046) in breast cancer patients than in controls, even after correcting the P value for multiple testing with 10,000 permutations (P = 0.0384). Although there was a trend for a higher proportion of the rs35593994 ‘A’ allele in patients with breast cancer compared to controls, the trend did not achieve statistical significance (P = 0.0798). No statistically significant associations were observed between other SNP (rs3181097, rs35593994, rs3181100, rs1181388, rs10932017, rs4673259, rs3769684, rs12693993, and rs3769686) and breast cancer risk in the study. The distribution of haplotypes defined in LD block 1 and LD block 2 did not statistically differ between patients and controls (**Table 4**).

**Table 2:** Genotyping of CD28 gene SNPs in breast cancer patients and controls.

SNP^1^	Minor^2^ (a)	Major^2^ (A)	Case^3^			Control^4^			*P* value for model of inheritance^5^			OR (95%CI)^6^
			AA	Aa	aa	AA	Aa	aa	Additive	Dominant	Recessive	
rs3181097	G	A	134	281	150	171	286	148	0.2037	0.0766	0.4132	1.267(0.975,1.648)
rs35593994	A	G	313	216	36	364	211	30	0.2142	0.0989	0.2952	1.216(0.964,1.534)
rs3181100	G	C	388	160	17	404	188	13	0.4181	0.4884	0.3523	0.917(0.717,1.172)
rs1181388	C	T	147	286	132	173	290	142	0.5658	0.3230	0.9652	1.139(0.880,1.473)
rs10932017	T	C	255	265	45	288	254	63	0.1440	0.3971	0.1482	1.104(0.878,1.390)
rs4673259	T	C	136	289	140	157	296	152	0.6993	0.4584	0.8915	1.105(0.848,1.441)
rs3769684	T	C	154	284	127	169	292	144	0.7760	0.7957	0.5917	1.034(0.800,1.337)
rs3116487	G	C	481	84	0	509	90	6	0.0608	0.6355	**0.0314**	0.926(0.674,1.273)
rs3116494	G	A	481	84	0	509	89	7	**0.0338**	0.6355	**0.0157**	0.926(0.674,1.273)
rs3116496	C	T	450	109	6	520	81	4	**0.0164**	**0.0042**	0.5353	1.563(1.150,2.126)
rs12693993	A	G	405	147	13	421	173	11	0.5425	0.4320	0.5605	0.904(0.703,1.163)

1 For SNP rs3119686, no GG genotype was detected in this study and the frequency of G allele is only 1.75%. Thus, only allelic *P* value was calculated between cases and controls in **Table 3**.

2 Minor allele ‘a’ and the major ‘A’ are shown in the table. ‘AA’, ‘Aa’, ‘aa’ represent a given variant for each SNP genotyped.

3 the number of cases in study cohort was 565.

4 the number of controls in study cohort was 605.

5 The *P* values were accessed using Plink and SPSS software under an additive model (AA vs. Aa vs. aa), dominant model (aa+Aa vs. AA), and recessive model (aa vs. Aa+AA) respectively. Significant values (*P*<0.05) are in bold.

6 Estimated odds ratio (OR) and 95% confidence interval (CI) above were assessed under an dominant model (aa+Aa vs. AA) using logistic regression with Plink 1.07.

**Table 3:** Alleles of the genotyped SNPs in CD28 gene.

SNP	Allele	Case, Control Ratios	Chi-square	*P* value^1^
rs3181097	A:G	549:581, 628:582	2.571	0.1088
rs35593994	G:A	842:288, 939:271	3.069	0.0798
rs3181100	G:C	194:936, 214:996	0.109	0.7415
rs1181388	T:C	580:550, 636:574	0.357	0.5503
rs10932017	C:T	775:355, 830:380	0.000	0.9954
rs4673259	C:T	561:569, 610:600	0.138	0.7107
rs3769684	T:C	538:592, 580:630	0.024	0.8757
rs3116487	G:C	84:1046, 102:1108	0.792	0.3734
rs3116494	G:A	84:1046, 103:1107	0.925	0.3362
rs3116496	T:C	1009:121, 1121:89	8.040	**0.0046** 2
rs12693993	A:G	173:957, 195:1015	0.286	0.5925
rs3769686	A:G	1110:20, 1189:21	0.004	0.9495

1 Allele data was analyzed using Haploview 4.1. Significant values (*p*<0.05) are in bold.

2 *P* = 0.0384 after correcting *P* value for multiple testing by Haploview 4.1 program using 10,000 permutations.

**Table 4:** Haplotype of the CD28 gene SNPs between cases and controls.

Haplotype block^1^ identified in CD28 gene	Haplotype	Freq.	Case, control ratios	Chi Square	*P* value	Permutation *P* value^2^
Block 1(rs3181097-rs35593994- rs3181100-rs1181388-rs10932017-rs4673259-rs3769684)	AGCTCCC	0.469	510.1:609.9, 577.9:620.1	1.689	0.1937	0.8582
	GACCTTT	0.208	243.0:877.0, 238.4:959.6	1.140	0.2857	0.9570
	GGGCCTT	0.133	145.7:974.3, 163.7:1034.3	0.216	0.6424	0.9998
	GGCCTTT	0.070	71.6:1048.4, 90.6:1107.4	1.227	0.2680	0.9463
	GGCTCTC	0.014	17.3:1102.7, 16.2:1181.8	0.151	0.6976	0.9999
	GGGCTTT	0.014	11.0:1109.0, 21.4:1176.6	2.714	0.0995	0.7081
	GGCTCCC	0.012	14.2:1105.8, 14.5:1183.5	0.016	0.8987	1.0000
Block 2(rs3116487- rs3116494)	CA	0.921	1046:84.0, 1107:101.0	0.689	0.4065	0.9906
	GG	0.079	84.0:1046.0, 101.0:1107.0	0.689	0.4065	0.9906

1 Blocks were constructed according to the values of D’ and r^2^ calculated from our own data. SNPs with the minor allele frequency less than 5% were excluded in the haplotype construction. The definition of the LD blocks were based on the method of Gabriel et al. with confidence limits for strong LD (upper, 0.85; lower, 0.70) and the confidence interval maximums for strong recombination (upper, 0.85) and for strong LD (upper, at least 0.8) in informative comparisons.

2 *P* values for haplotypes in the two blocks were permutated 10,000 times by Haploview 4.1 program.

### Association between CD28 Gene Variants and Clinical Features of Breast Cancer

The clinical features of 565 sporadic breast cancer patients are summarized in **Table 1**, including tumor type, tumor size, ER, PR, C-erbB2, and P53 status, and lymph node metastasis condition. **Tables S1, S2, S3, S4, S5, S6** list the data involved in the clinical features analysis. A significant association was found between rs3116487/rs3116494 (D’ = 1, r^2^ = 0.99) and ER status at the single SNP level (allelic P = 0.013, dominant P = 0.0078). Similar to its role in the ER, rs3116487/rs3116494 (D’ = 1, r^2^ = 0.99) was also observed to be associated with C-erbB2 status in breast cancer patients (allelic P = 0.0247, dominant P = 0.0198). We further analyzed the association between haplotypes identified and clinical features using Haploview software. LD Block 2 was associated with ER and C-erbB2 status, which was in accordance with the results at the single SNP level. Additionally, a moderate association was found between LD Block 1 G_rs3181097_G_rs35593994_C_rs3181100_C_rs1181388_C_rs10932017_T_rs4673259_T_rs3769684_ haplotype and ER status (P = 0.0309). The G_rs3181097_G_rs35593994_C_rs3181100_T_rs1181388_C_rs10932017_T_rs4673259_C_rs3769684_ haplotype had a higher prevalence in C-erbB2 positive patients compared to C-erbB2 negative ones (P = 0.0242). The distribution of other haplotypes did not differ between patients and controls. No statistically significant relationships were observed in regard to histological grade (data not shown), lymph node metastasis, or PR and P53 status.

## Discussion

The etiology and pathogenesis of breast cancer depend on multiple factors. Understanding of the patient’s genetic background helps to optimize the approaches for breast cancer prevention and treatment. Previous studies have extensively examined the association of CTLA-4 polymorphisms and malignancies [29], [30]. As a homolog of CTLA-4, CD28 may also contribute to the development of cancer. In this case-control study, we genotyped SNPs that completely span the CD28 gene region, and we classified the association between CD28 gene variants and sporadic breast cancer in a Chinese Han population in Northeast China.

There was a difference in the rs3116496 polymorphism of the CD28 gene between breast cancer patients and controls in a Chinese Han population. The rs3116496 CC+CT genotype and ‘C’ allele frequencies were higher in breast cancer patients compared with controls, suggesting that the rs3116496 CC+CT genotype and ‘C’ allele may contribute to breast carcinogenesis. The rs3116496 SNP is located at position IVS3+17 of the third intron of the CD28 gene. It is difficult to explain the exact biological function of intronic SNPs. However, the potential explanations may be as follows. The intronic SNP rs3116496 is located near the splice acceptor site [23], [26], where mutations may induce aberrant splicing due to disruption of the splice site [31]. This polymorphism probably alters the expression of the CD28 protein by influencing mRNA splicing, and eventually, leads to immune alteration.

Alternative splicing in CD28 pre-mRNA gives rise to different mRNA isoforms, leading to soluble and membrane-bound isoforms of expressed CD28 protein [32], [33], [34]. Balance of these putative polypeptides, with different physiologic roles, [34] is important to immune homeostasis. It is likely that intronic SNPs in the CD28 gene, e.g. rs3116496, lead to the unbalanced expression of various CD28 protein isoforms by aberrant splicing, which leads to altered immune function. In line with this hypothesis, a report comparing sCD28 expression of each rs3116496 genotype in rheumatoid arthritis patients found higher sCD28 levels in TT carriers than in those with the TC genotype [21]. Further studies of the potential mechanisms underlying intronic polymorphisms, on CD28 pre-mRNA splicing, will therefore be interesting.

rs3116496 is probably only one marker in linkage disequilibrium with other true susceptible variants. The relationship between the CD28 rs3116496 polymorphisms and susceptibility to various diseases, including cancer, has been previously investigated. Bechet’s disease [22], rheumatoid arthritis [21], and cervical cancer [23], [24], [25], [26], [27] are all known to be associated with the rs3116496 polymorphism. It was reported that the rs3116496 TT genotype was associated with a low risk of cervical cancer in a Chinese Han and in a Swedish population, respectively [23], [24]. Guzman et al. [26] observed that the rs3116496 TT genotype, combined with IFN+847AA, increased cervical cancer risk in a Brazilian population. Pawlak et al. [25] demonstrated that this SNP was not associated with cervical squamous cell carcinoma. These conflicting observations in cervical cancer, among different ethnic groups, are interesting. Genetic background and environmental factors are different among populations. Every population shares their own linkage disequilibrium pattern. Accordingly, a functional SNP may be in linkage disequilibrium with distinct markers in different ethnic groups. Thus, rs3116496 may be only a marker linked to the real functional variant in the studied population, with no biological function itself at all. Nevertheless, we cannot deny the possibility that functional rs3116496, together with other uncovered functional variants in linkage disequilibrium, synergistically influence disease risk. Epidemiological studies with ethnically diverse populations need to evaluate this hypothesis.

There was a trend toward higher proportion of rs35593994 ‘A’ allele and genotype (AA+AG) in the dominant genetic model, in patients with breast cancer, compared with controls. Non-coding SNPs may disrupt transcription factor binding sites, splice sites, and other functional sites at the transcription level. The rs35593994 SNP is located in the promoter region of the CD28 gene. A search for a TFBS (transcription factor binding site) using the TRANSFAC program [35] revealed that rs35593994 G/A alleles may influence gene transcription. Compared with rs35593994 ‘G’, rs35593994 ‘A’ may promote transcription of the CD28 gene by the presence of a binding site available for the CCAAT enhancer-binding protein, but not GFI1 (which functions as a transcriptional repressor) [36]. The immune alteration generated by this potentially functional SNP probably contributes to disease risk. However, the association between this SNP and breast cancer risk did not achieve statistical significance in our study.

In the clinical features analysis, associations were found between rs3116487/rs3116494 (D’ = 1, r^2^ = 0.99) and the status of C-erbB2 and ER in breast patients. Similar to rs3116496, rs3116487 was located in intron 1 and rs3116494 was located in intron 2. They could contribute to splicing events which play roles in CD28 signaling and T lymphocyte activation. It has been well established that routine clinical management of breast cancer depends on clinicopathological factors, such as ER and C-erbB2 expression. Steroid hormone receptors play an important role in disease-free (DFS) and overall survival (OS) of breast cancer patients, and are considered predictive markers of endocrine therapy [37]. Breast cancer patients with a complete lack of ER expression do not benefit from endocrine therapies [38], [39]. C-erbB2 expression is accepted as a significant prognostic factor for DFS and OS. Overexpression of C-erbB2 in breast cancer patients is associated with a worse prognosis and a higher recurrence rate [39], [40]. C_rs3116487_A_rs3116494_ had a higher prevalence in ER positive subgroups, and a lower prevalence in C-erbB2 positive ones. C_rs3116487_A_rs3116494_ was probably a marker for good prognosis in breast cancer patients. The exact biological function of these SNPs on the expression of ER and C-erbB2 in breast cancer is not known. From the evidence of this case-control study we can just conclude that the two SNPs may be potential markers to predict prognosis of breast cancer patients.

In the haplotype analysis, LD among polymorphic loci was assessed using Haploview software. Only two LD blocks were identified. LD block 1 G_rs3181097_G_rs35593994_C_rs3181100_C_rs1181388_C_rs10932017_T_rs4673259_T_rs3769684_ haplotype was associated with ER status, while block 1 G_rs3181097_G_rs35593994_C_rs3181100_T_rs1181388_C_rs10932017_T_rs4673259_C_rs3769684_ haplotype had a higher prevalence in the C-erbB2 positive subgroup. LD Block 2 was associated with ER and C-erbB2 status, in accordance with the results at the single SNP level. These results indicated that rs3116487/rs3116494 (D’ = 1, r^2^ = 0.99) might be meaningful in the pathology of breast cancer, as well as potential markers to predict prognosis of breast cancer patients.

Taken together, the current study shows that polymorphisms of the CD28 gene region may affect susceptibility to sporadic breast cancer risk in Chinese Han women. This study provides the first evidence for the involvement of the human CD28 gene in breast cancer. The clinical features analysis in this study revealed an association between CD28 gene polymorphisms and prognostic factors in breast cancer, including ER and C-erbB2 status. However, the basic functions of CD28 gene mutations remain to be further elucidated.

## Materials and Methods

### Ethics Statement

This study was conducted in Heilongjiang Province in northeast of China. Before the research was conducted, ethical board approval from the Third Affiliated Hospital of Harbin Medical University was obtained, and all of the volunteers provided written informed consent.

### Subjects

All sporadic breast cancer patients were recruited from the Third Affiliated Hospital of Harbin Medical University, Heilongjiang Province, China. Patients’ clinical information included tumor type, tumor size, lymph node metastasis, human epidermal growth factor receptor 2 (C-erbB2), estrogen receptor (ER), progesterone receptor (PR) and tumor protein 53 (P53) status (**Table 1**). Our study consisted of 565 patients (mean age 49.6±10.3 years). All patients were diagnosed based on surgical and pathological findings. For comparison, 605 healthy controls (mean age 49.0±9.8 years) were recruited randomly from community volunteers. The healthy controls had no history of personal or familial malignancy or autoimmune disorders, and were frequency matched to patients by age.

### Preparation of Genomic DNA

Lymphocytes were separated from 5 ml of anticoagulated whole blood by centrifugation. Genomic DNA was extracted using the Universal Genomic DNA Extraction Kit Ver. 3.0 (TaKaRa, Japan), following the manufacturer’s protocol.

### SNP Selection

SNPs were selected using the HapMap database. Thirteen haplotype-tagging SNPs [rs1879877, rs3181097, rs3181100, rs2140148, rs1181388, rs10932017, rs4673259, rs3769684, rs3116486 (replaced by rs3116487 in this study with r^2^ = 1), rs3116494, rs3116496, rs12693993, and rs3769686] from the CD28 gene were selected from the HapMap database using Haploview software 4.1, with pair-wise r^2^<0.8 for each SNP pair and minor allele frequencies >5%. In addition to the SNPs mentioned above, the SNP rs35593994, on which the HapMap database has no information, was selected in this study because it has been reported to be potentially functional. All selected SNPs were validated using extensive database searches (http://www.ensemble.org,http://ncbi.nlm.nih.gov/SNP). In total, 14 SNPs were selected to span the entire CD28 gene region.

### Genotyping

All 14 SNPs were genotyped using the polymerase chain reaction-restriction fragment length polymorphism (PCR-RFLP) assay. The accuracy of genotyping results were confirmed using direct sequencing in random samples. The primers and restriction enzymes for CD28 PCR-RFLP genotyping are shown in **Table S7**. The polymorphic regions were amplified by PCR using a T-Gradient Thermoblock PCR System (BioRad, USA) in a 25 µl-reaction solution containing 0.3 µg genomic DNA, 2.5 µl 10× PCR buffer (Mg^2+^ plus), 2.0 µl dNTP mixture (TaKaRa, Japan), 2.5 U TaqDNA polymerase (TaKaRa, Japan), and 0.2 µl of each primer (10 umol/L) (Invitrogen, China). The PCR products of each SNP were digested with restriction enzymes (NEB, USA), following the manufacturer’s instructions (**Table S7**), and isolated using 2% (for rs3181097, rs3181100, rs2140148, rs10932017, rs4673259, rs3769684, rs3116487, rs12693993, rs3769686, and rs35593994) and 3% (for rs1879877, rs1181388, rs3116494, and rs3116496) agarose gel electrophoresis.

### Statistical Analysis

The deviation from Hardy-Weinberg equilibrium (HWE) was determined using a goodness-of-fit chi-squared test to compare the observed genotype frequencies with the expected frequencies, from the healthy controls. The polymorphisms were excluded if they deviated from HWE, or if missing data composed more than 10% of the total data. Using different models of inheritance (additive, dominant, recessive), the genotype frequencies of the subjects were analyzed using the chi-squared test and Fisher’s exact test. Estimated odds ratio (OR) and 95% confidence interval (CI) were assessed using logistic regression with Plink software. To determine the significance with corrections for multiple testing biases, we ran 10,000 permutations using Haploview to determine the P value. All data were analyzed using SPSS (version 17.0), Plink (version 1.07) (http://pngu.mgh.harvard.edu/~purcell/plink), and Haploview (version 4.1) (http://www.broad.mit.edu/mpg/haploview/). The threshold for significance was P<0.05, and the relative risks associated with haplotypes were estimated as odds ratios (ORs), with 95% confidence intervals (CIs).

Haplotype patterns were generated using the algorithm described in Haploview [28], which constructs haplotypes based on the D’ measure of linkage disequilibrium and a LOD score as a measure of significance. The pair-wise linkage disequilibrium for a genotyped SNP was confirmed using r^2^ values [41], [42]. SNPs with a minor allele frequency of less than 5% were excluded in our haplotype constructions. Haplotype block definitions were based on the method of Gabriel et al. [28], with confidence limits for strong LD (upper, 0.85; lower, 0.70), and confidence interval maximums for strong recombination (upper, 0.85) and strong LD (upper, at least 0.8) in informative comparisons.

## Supporting Information

Figure S1 **Haplotypic architecture in CD28.** Haplotype LD block structure across the CD28 gene was generated from 565 breast cancer patients and 605 healthy individuals in a Chinese Han population using Haploview software. **(A)** LD prime chart from Haploview that summarizes the LD patterns is shown. The numbers in each box represent the LD value between the adjacent SNPs. **(B)** r^2^ prime chart from Haploview that summarizes the r^2^ patterns is shown. The numbers in each box represent the r^2^ value between the adjacent SNPs. The physical representations of the SNP positions in **(A, B)** have been colored to represent the LD between the adjacent SNPs, according to the standard Haploview software color scheme: LOD>2 and D' = 1, red; LOD>2 and D'<1, shades of pink/red; LOD<2 and D' = 1, blue; LOD<2 and D'<1, white. The definition of the LD blocks were based on the method of Gabriel et al. with confidence limits for strong LD (upper, 0.85; lower, 0.70), and confidence interval maximums for strong recombination (upper, 0.85) and strong LD (upper, at least 0.8), in informative comparisons. In order to help keep the display uncluttered, D prime values of 1.0 were never shown (the box is empty). The strongest LD shown above was between rs3116487 and rs3116494 (D’ = 1, r^2^ = 0.99). Two LD blocks were identified from the CD28 gene are presented. Block 1 covered 14 kb, and block 2 about 5 kb. SNP rs3119686, with minor allele frequency of only 1.75%, was excluded from the haplotype analysis.
(TIF)

Figure S2 **Distribution of haplotypes in the case group, the control group and the whole group.** Haplotypes with frequency more than 1% are shown in the case group, the control group, and the whole group. Seven haplotypes in block 1 and two haplotypes in block 2 are shown above. LD block1-A_rs3181097_G_rs35593994_C_rs3181100_T_rs1181388_C_rs10932017_C_rs4673259_C_rs3769684_ and LD block 2- C_rs3116487_A_rs3116494_ are the most common haplotypes in each subgroup.
(TIF)

Table S1 **Relationship between ER status in breast cancer patients and variants detected in the CD28 gene.** ^1^ER information of 484 breast cancer patients was available in the study with 282 (49.91%) positive and 202 (35.75%) negative ones. ^2^The *P* values were accessed using Plink and SPSS software under an additive model (AA vs. Aa vs. aa), dominant model (aa+Aa vs. AA), and recessive model (aa vs. aA+AA) respectively. Significant values (*P*<0.05) are in bold.
(DOC)

Table S2 **Relationship between PR status in breast cancer patients and variants detected in the CD28 gene.** ^1^PR information of 482 breast cancer patients was available in the study with 346 (61.24%) positive and 136 (24.07%) negative ones. ^2^The *P* values were accessed using Plink and SPSS software under an additive model (AA vs. Aa vs. aa), dominant model (aa+Aa vs. AA), and recessive model (aa vs. aA+AA) respectively. Significant values (*P*<0.05) are in bold.
(DOC)

Table S3 **Relationship between C-erbB2 status in breast cancer patients and variants detected in the CD28 gene.** ^1^C-erbB2 information of 479 breast cancer patients was available in the study with 187 (33.10%) positive and 292 (51.68%) negative ones. ^2^The *P* values were accessed using Plink and SPSS software under an additive model (AA vs. Aa vs. aa), dominant model (aa+Aa vs. AA), and recessive model (aa vs. aA +AA) respectively. Significant values (*P*<0.05) are in bold.
(DOC)

Table S4 **Relationship between P53 status in breast cancer patients and variants detected in the CD28 gene.** ^1^P53 information of 467 breast cancer patients was available in the study with 146 (25.84%) positive and 321 (56.81%) negative ones. ^2^The *P* values were accessed using Plink and SPSS software under an additive model (AA vs. Aa vs. aa), dominant model (aa+Aa vs. AA), and recessive model (aa vs. aA+AA) respectively. Significant values (*P*<0.05) are in bold.
(DOC)

Table S5 **Relationship between lymph node involvement status in breast cancer patients and variants detected in the CD28 gene.** ^1^LN, lymph node. ^2^LN involvement information of 543 breast cancer patients was available in the study with 235 (41.59%) positive and 310 (54.87%) negative ones. ^3^The *P* values were accessed using Plink and SPSS software under an additive model (AA vs. Aa vs. aa), dominant model (aa+Aa vs. AA), and recessive model (aa vs. aA+AA) respectively. Significant values (*P*<0.05) are in bold.
(DOC)

Table S6 **Association between tumor size and polymorphisms.** ^1^466 tumor size information in detail of breast cancer patients were available. ^2^I: Tumor with the diameter less than 2 cm (0<tumor size< = 2), number = 187.^ 3^II: Tumor with the diameter of 2 to 5 cm (2<tumor size< = 5), number = 252. ^4^III: Tumor with the diameter more than 5 (tumor size>5), number = 27.
(DOC)

Table S7 **Genotyping information for 14 selected SNPs.** ^1^NEB Cutter, names of restriction enzymes purchased from the NEB biolabs company.
(DOC)
