# Supplementary figures and images for: Investigation of CD28 Gene Polymorphisms in Patients with Sporadic Breast Cancer in a Chinese Han Population in Northeast China
Source: PLoS One. 2012 Oct 25;7(10):e48031. doi: 10.1371/journal.pone.0048031 (PMC3485049; doi:10.1371/journal.pone.0048031)

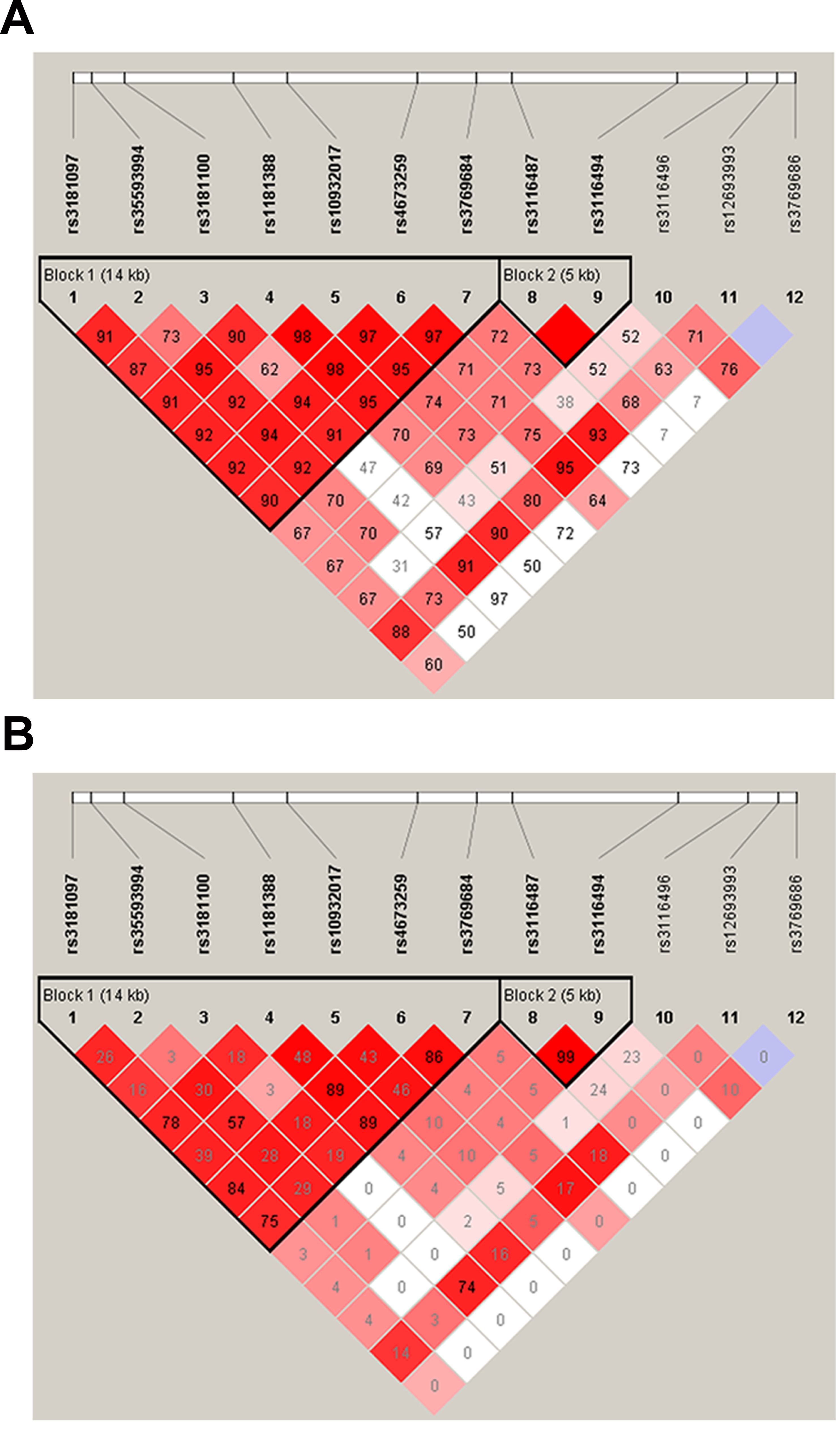

Supplement: Figure S1 — Haplotypic architecture in CD28. Haplotype LD block structure across the CD28 gene was generated from 565 breast cancer patients and 605 healthy individuals in a Chinese Han population using Haploview software. (A) LD prime chart from Haploview that summarizes the LD patterns is shown. The numbers in each box represent the LD value between the adjacent SNPs. (B) r2 prime chart from Haploview that summarizes the r2 patterns is shown. The numbers in each box represent the r2 value between the adjacent SNPs. The physical representations of the SNP positions in (A, B) have been colored to represent the LD between the adjacent SNPs, according to the standard Haploview software color scheme: LOD>2 and D' = 1, red; LOD>2 and D'<1, shades of pink/red; LOD<2 and D' = 1, blue; LOD<2 and D'<1, white. The definition of the LD blocks were based on the method of Gabriel et al. with confidence limits for strong LD (upper, 0.85; lower, 0.70), and confidence interval maximums for strong recombination (upper, 0.85) and strong LD (upper, at least 0.8), in informative comparisons. In order to help keep the display uncluttered, D prime values of 1.0 were never shown (the box is empty). The strongest LD shown above was between rs3116487 and rs3116494 (D’ = 1, r2 = 0.99). Two LD blocks were identified from the CD28 gene are presented. Block 1 covered 14 kb, and block 2 about 5 kb. SNP rs3119686, with minor allele frequency of only 1.75%, was excluded from the haplotype analysis. (TIF) [file pone.0048031.s001.tif]

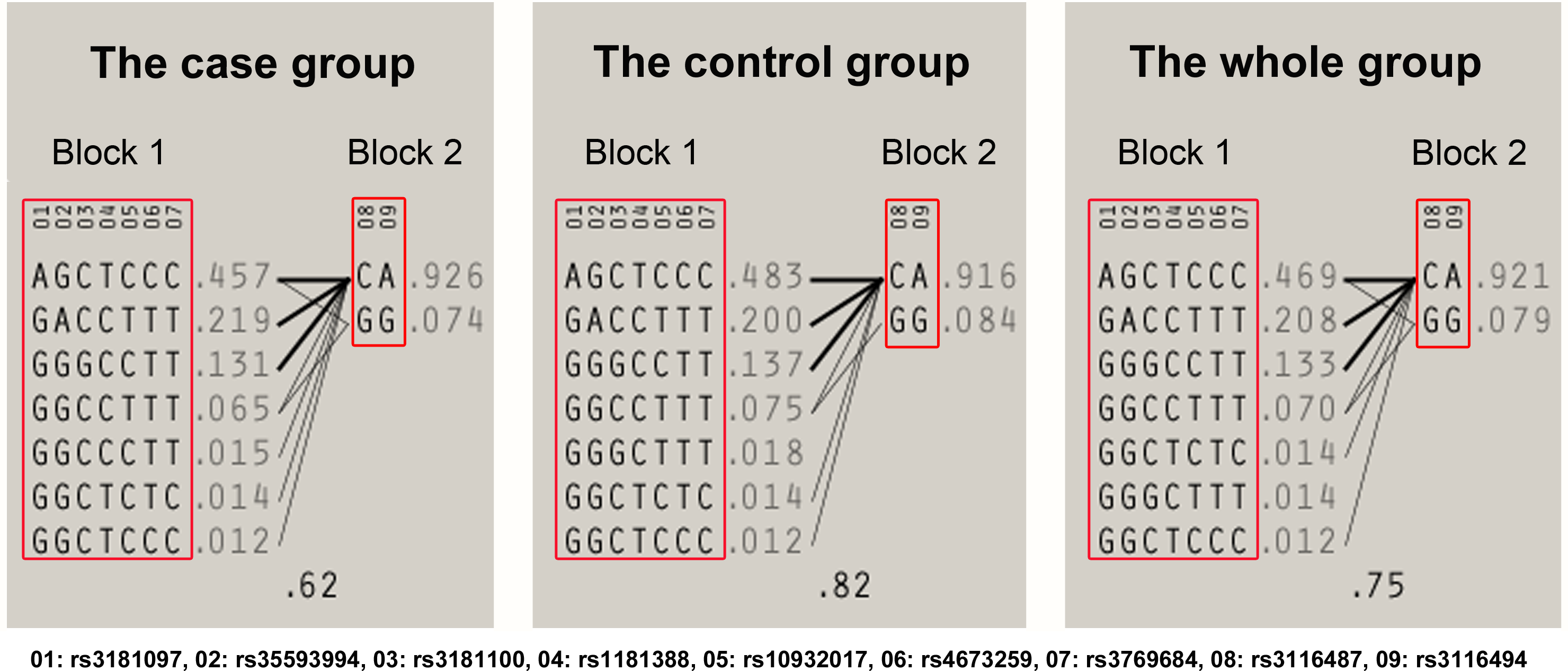

Supplement: Figure S2 — Distribution of haplotypes in the case group, the control group and the whole group. Haplotypes with frequency more than 1% are shown in the case group, the control group, and the whole group. Seven haplotypes in block 1 and two haplotypes in block 2 are shown above. LD block1-Ars3181097Grs35593994Crs3181100Trs1181388Crs10932017Crs4673259Crs3769684 and LD block 2- Crs3116487Ars3116494 are the most common haplotypes in each subgroup. (TIF) [file pone.0048031.s002.tif]
