# Supplementary material for: Investigation of CD28 Gene Polymorphisms in Patients with Sporadic Breast Cancer in a Chinese Han Population in Northeast China
Source: PLoS One. 2012 Oct 25;7(10):e48031. doi: 10.1371/journal.pone.0048031 (PMC3485049; doi:10.1371/journal.pone.0048031)
Supplement: Table S1 — Relationship between ER status in breast cancer patients and variants detected in the CD28 gene. 1ER information of 484 breast cancer patients was available in the study with 282 (49.91%) positive and 202 (35.75%) negative ones. 2The P values were accessed using Plink and SPSS software under an additive model (AA vs. Aa vs. aa), dominant model (aa+Aa vs. AA), and recessive model (aa vs. aA+AA) respectively. Significant values (P<0.05) are in bold. (DOC) [file pone.0048031.s003.doc]

**Table S1 Relationship between ER status in breast cancer patients and variants detected in the CD28 gene**

| Relationship with ER^1^ status on SNP level | | | | | | |  | Relationship with ER^1^ status on Haplotype level | | | | |
| --- | --- | --- | --- | --- | --- | --- | --- | --- | --- | --- | --- | --- |
| SNP ID | "a"* | "A"* | Model^2^ | Positive | Negative | *P* value |  | Haplotype | Freq. | Positive, negative ratios | Chi-square | *P* value |
| rs3181097 | A | G | Additive | 62/147/73 | 49/95/58 | 0.5423 |  |  |  |  |  |  |
| rs3181097 | A | G | Allelic | 271/293 | 193/211 | 0.9321 |  | BLOCK1 |  |  |  |  |
| rs3181097 | A | G | Dominant | 209/73 | 144/58 | 0.4901 |  | BLOCK1-AGCTCCC | 0.449 | 257.3:306.7,177.7:226.3 | 0.254 | 0.6143 |
| rs3181097 | A | G | Recessive | 62/220 | 49/153 | 0.5577 |  | BLOCK1-GACCTTT | 0.223 | 118.6:445.4,97.7:306.3 | 1.363 | 0.2430 |
| rs35593994 | A | G | Additive | 17/108/157 | 16/78/108 | 0.6956 |  | BLOCK1-GGGCCTT | 0.138 | 79.0:485.0,54.6:349.4 | 0.049 | 0.8243 |
| rs35593994 | A | G | Allelic | 142/422 | 110/294 | 0.4734 |  | BLOCK1-GGCCTTT | 0.058 | 35.3:528.7,20.5:383.5 | 0.601 | 0.4383 |
| rs35593994 | A | G | Dominant | 125/157 | 94/108 | 0.6303 |  | **BLOCK1-GGCCCTT** | 0.016 | 13.0:551.0,2.2:401.8 | 4.660 | **0.0309** |
| rs35593994 | A | G | Recessive | 17/265 | 16/186 | 0.4154 |  | BLCOK1-GGCTCCC | 0.015 | 7.0:557.0,7.1:396.9 | 0.455 | 0.4998 |
| rs3181100 | G | C | Additive | 11/72/199 | 4/66/132 | 0.1370 |  | BLOCK1-GGCTCTC | 0.013 | 6.5:557.5,6.0:398.0 | 0.194 | 0.6598 |
| rs3181100 | G | C | Allelic | 94/470 | 74/330 | 0.5038 |  |  |  |  |  |  |
| rs3181100 | G | C | Dominant | 83/199 | 70/132 | 0.2232 |  | BLOCK 2 |  |  |  |  |
| rs3181100 | G | C | Recessive | 11/271 | 4/198 | 0.2293 |  | **BLOCK2-CA** | 0.935 | 537.0:27.0,368.0:36.0 | 6.578 | **0.0103** |
| rs1181388 | C | T | Additive | 63/151/68 | 52/96/54 | 0.4211 |  | **BLOCK2-GG** | 0.065 | 27.0:537.0,36.0:368.0 | 6.578 | **0.0103** |
| rs1181388 | C | T | Allelic | 277/287 | 200/204 | 0.9044 |  |  |  |  |  |  |
| rs1181388 | C | T | Dominant | 214/68 | 148/54 | 0.5128 |  |  |  |  |  |  |
| rs1181388 | C | T | Recessive | 63/219 | 52/150 | 0.3858 |  |  |  |  |  |  |
| rs10932017 | T | C | Additive | 19/130/133 | 19/95/88 | 0.4909 |  |  |  |  |  |  |
| rs10932017 | T | C | Allelic | 168/396 | 133/271 | 0.2990 |  |  |  |  |  |  |
| rs10932017 | T | C | Dominant | 149/133 | 114/88 | 0.4332 |  |  |  |  |  |  |
| rs10932017 | T | C | Recessive | 19/263 | 19/183 | 0.2818 |  |  |  |  |  |  |
| rs4673259 | C | T | Additive | 64/153/65 | 49/95/58 | 0.2457 |  |  |  |  |  |  |
| rs4673259 | C | T | Allelic | 281/283 | 193/211 | 0.5292 |  |  |  |  |  |  |
| rs4673259 | C | T | Dominant | 217/65 | 144/58 | 0.1582 |  |  |  |  |  |  |
| rs4673259 | C | T | Recessive | 64/218 | 49/153 | 0.6887 |  |  |  |  |  |  |
| rs3769684 | T | C | Additive | 61/146/75 | 50/98/54 | 0.6878 |  |  |  |  |  |  |
| rs3769684 | T | C | Allelic | 268/296 | 198/206 | 0.6468 |  |  |  |  |  |  |
| rs3769684 | T | C | Dominant | 207/75 | 148/54 | 0.9732 |  |  |  |  |  |  |
| rs3769684 | T | C | Recessive | 61/221 | 50/152 | 0.4206 |  |  |  |  |  |  |
| rs3116487 | G | C | Allelic | 27/537 | 36/368 | **0.0103** |  |  |  |  |  |  |
| rs3116487 | G | C | Dominant | 27/255 | 36/166 | **0.0078** |  |  |  |  |  |  |
| rs3116494 | G | A | Allelic | 27/537 | 36/368 | **0.0103** |  |  |  |  |  |  |
| rs3116494 | G | A | Dominant | 27/255 | 36/166 | **0.0078** |  |  |  |  |  |  |
| rs3116496 | C | T | Additive | 1/47/234 | 4/41/157 | 0.1093 |  |  |  |  |  |  |
| rs3116496 | C | T | Allelic | 49/515 | 49/355 | 0.0801 |  |  |  |  |  |  |
| rs3116496 | C | T | Dominant | 48/234 | 45/157 | 0.1478 |  |  |  |  |  |  |
| rs3116496 | C | T | Recessive | 1/281 | 4/198 | 0.1660 |  |  |  |  |  |  |
| rs12693993 | A | G | Additive | 9/71/202 | 3/57/142 | 0.4019 |  |  |  |  |  |  |
| rs12693993 | A | G | Allelic | 89/475 | 63/341 | 0.9375 |  |  |  |  |  |  |
| rs12693993 | A | G | Dominant | 80/202 | 60/142 | 0.7496 |  |  |  |  |  |  |
| rs12693993 | A | G | Recessive | 9/273 | 3/199 | 0.2339 |  |  |  |  |  |  |
| rs3769686 | G | A | Allelic | 11/553 | 6/398 | 0.5869 |  |  |  |  |  |  |
| rs3769686 | G | A | Dominant | 11/271 | 6/196 | 0.5835 |  |  |  |  |  |  |

^1^ER information of 484 breast cancer patients was available in the study with 282 (49.91%) positive and 202 (35.75%) negative ones.

^2^The *P* values were accessed using Plink and SPSS software under an additive model (AA vs. Aa vs. aa), dominant model (aa+Aa vs. AA), and recessive model (aa vs. aA+AA) respectively. Significant values (*P* <0.05) are in bold.

*Minor allele ‘a’ and the major ‘A’ are shown in the table. ‘AA’, ‘Aa’, ‘aa’ represent a given variant for each SNP genotyped.
