# Supplementary material for: Investigation of CD28 Gene Polymorphisms in Patients with Sporadic Breast Cancer in a Chinese Han Population in Northeast China
Source: PLoS One. 2012 Oct 25;7(10):e48031. doi: 10.1371/journal.pone.0048031 (PMC3485049; doi:10.1371/journal.pone.0048031)
Supplement: Table S2 — Relationship between PR status in breast cancer patients and variants detected in the CD28 gene. 1PR information of 482 breast cancer patients was available in the study with 346 (61.24%) positive and 136 (24.07%) negative ones. 2The P values were accessed using Plink and SPSS software under an additive model (AA vs. Aa vs. aa), dominant model (aa+Aa vs. AA), and recessive model (aa vs. aA+AA) respectively. Significant values (P<0.05) are in bold. (DOC) [file pone.0048031.s004.doc]

**Table S2 Relationship between PR status in breast cancer patients and variants detected in the CD28 gene**

| Relationship with PR^1^ status on SNP level | | | | | | |  | Relationship with PR^1^ status on Haplotype level | | | | |
| --- | --- | --- | --- | --- | --- | --- | --- | --- | --- | --- | --- | --- |
| SNP ID | "a"* | "A"* | Model^2^ | Positive | Negative | *P* value |  | Haplotype | Freq. | Positive, negative ratios | Chi-square | *P* value |
| rs3181097 | A | G | Additive | 80/172/94 | 30/70/36 | 0.9389 |  |  |  |  |  |  |
| rs3181097 | A | G | Allelic | 332/360 | 130/142 | 0.9592 |  | BLOCK1 |  |  |  |  |
| rs3181097 | A | G | Dominant | 252/94 | 100/36 | 0.8767 |  | BLOCK1-AGCTCCC | 0.449 | 316.0:376.0,117.0:155.0 | 0.551 | 0.4580 |
| rs3181097 | A | G | Recessive | 80/266 | 30/106 | 0.8025 |  | BLOCK1-GACCTTT | 0.223 | 151.9:540.1,63.5:208.5 | 0.217 | 0.6412 |
| rs35593994 | A | G | Additive | 23/132/191 | 10/53/73 | 0.9373 |  | BLOCK1-GGGCCTT | 0.138 | 98.0:594.0,34.5:237.5 | 0.356 | 0.5509 |
| rs35593994 | A | G | Allelic | 178/514 | 73/199 | 0.7224 |  | BLOCK1-GGCCTTT | 0.058 | 41.7:650.3,14.1:257.9 | 0.249 | 0.6175 |
| rs35593994 | A | G | Dominant | 155/191 | 63/73 | 0.7620 |  | BLOCK1-GGCCCTT | 0.016 | 12.1:679.9,3.2:268.8 | 0.426 | 0.5139 |
| rs35593994 | A | G | Recessive | 23/323 | 10/126 | 0.7825 |  | BLCOK1-GGCTCCC | 0.015 | 7.2:684.8,6.9:265.1 | 3.080 | 0.0793 |
| rs3181100 | G | C | Additive | 14/95/237 | 1/42/93 | 0.1464 |  | BLOCK1-GGCTCTC | 0.013 | 8.5:683.5,4.0:268.0 | 0.096 | 0.7565 |
| rs3181100 | G | C | Allelic | 123/569 | 44/228 | 0.5552 |  |  |  |  |  |  |
| rs3181100 | G | C | Dominant | 109/237 | 43/93 | 0.9805 |  | BLOCK 2 |  |  |  |  |
| rs3181100 | G | C | Recessive | 14/332 | 1/135 | 0.7840 |  | BLOCK2-CA | 0.935 | 649.0:43.0,252.0:20.0 | 0.415 | 0.5196 |
| rs1181388 | C | T | Additive | 83/174/89 | 31/73/32 | 0.7927 |  | BLOCK2-GG | 0.065 | 43.0:649.0,20.0:252.0 | 0.415 | 0.5196 |
| rs1181388 | C | T | Allelic | 340/352 | 135/137 | 0.8890 |  |  |  |  |  |  |
| rs1181388 | C | T | Dominant | 257/89 | 104/32 | 0.6173 |  |  |  |  |  |  |
| rs1181388 | C | T | Recessive | 83/263 | 31/105 | 0.7812 |  |  |  |  |  |  |
| rs10932017 | T | C | Additive | 27/161/158 | 11/63/62 | 0.9945 |  |  |  |  |  |  |
| rs10932017 | T | C | Allelic | 215/477 | 85/187 | 0.9565 |  |  |  |  |  |  |
| rs10932017 | T | C | Dominant | 188/158 | 74/62 | 0.9879 |  |  |  |  |  |  |
| rs10932017 | T | C | Recessive | 27/319 | 11/125 | 0.9168 |  |  |  |  |  |  |
| rs4673259 | C | T | Additive | 83/174/89 | 29/74/33 | 0.7037 |  |  |  |  |  |  |
| rs4673259 | C | T | Allelic | 340/352 | 132/140 | 0.8660 |  |  |  |  |  |  |
| rs4673259 | C | T | Dominant | 257/89 | 103/33 | 0.7404 |  |  |  |  |  |  |
| rs4673259 | C | T | Recessive | 83/263 | 29/107 | 0.5330 |  |  |  |  |  |  |
| rs3769684 | T | C | Additive | 82/168/96 | 28/76/32 | 0.3494 |  |  |  |  |  |  |
| rs3769684 | T | C | Allelic | 332/360 | 132/140 | 0.8772 |  |  |  |  |  |  |
| rs3769684 | T | C | Dominant | 250/96 | 104/32 | 0.3455 |  |  |  |  |  |  |
| rs3769684 | T | C | Recessive | 82/264 | 28/108 | 0.4639 |  |  |  |  |  |  |
| rs3116487 | G | C | Allelic | 43/649 | 20/252 | 0.5196 |  |  |  |  |  |  |
| rs3116487 | G | C | Dominant | 43/303 | 20/116 | 0.5043 |  |  |  |  |  |  |
| rs3116494 | G | A | Allelic | 43/649 | 20/252 | 0.5196 |  |  |  |  |  |  |
| rs3116494 | G | A | Dominant | 43/303 | 20/116 | 0.5043 |  |  |  |  |  |  |
| rs3116496 | C | T | Additive | 3/60/283 | 2/28/106 | 0.5453 |  |  |  |  |  |  |
| rs3116496 | C | T | Allelic | 66/626 | 32/240 | 0.3031 |  |  |  |  |  |  |
| rs3116496 | C | T | Dominant | 63/283 | 30/106 | 0.3350 |  |  |  |  |  |  |
| rs3116496 | C | T | Recessive | 3/343 | 2/134 | 0.6241 |  |  |  |  |  |  |
| rs12693993 | A | G | Additive | 11/90/245 | 1/37/98 | 0.2987 |  |  |  |  |  |  |
| rs12693993 | A | G | Allelic | 112/580 | 39/233 | 0.4777 |  |  |  |  |  |  |
| rs12693993 | A | G | Dominant | 101/245 | 38/98 | 0.7852 |  |  |  |  |  |  |
| rs12693993 | A | G | Recessive | 11/335 | 1/135 | 0.1930 |  |  |  |  |  |  |
| rs3769686 | G | A | Allelic | 12/680 | 5/267 | 0.9120 |  |  |  |  |  |  |
| rs3769686 | G | A | Dominant | 12/334 | 5/131 | 1.0000 |  |  |  |  |  |  |

^1^PR information of 482 breast cancer patients was available in the study with 346 (61.24%) positive and 136 (24.07%) negative ones.

^2^The *P* values were accessed using Plink and SPSS software under an additive model (AA vs. Aa vs. aa), dominant model (aa+Aa vs. AA), and recessive model (aa vs. aA +AA) respectively. Significant values (*P* <0.05) are in bold.

*Minor allele ‘a’ and the major ‘A’ are shown in the table. ‘AA’, ‘Aa’, ‘aa’ represent a given variant for each SNP genotyped.
