# Supplementary material for: Investigation of CD28 Gene Polymorphisms in Patients with Sporadic Breast Cancer in a Chinese Han Population in Northeast China
Source: PLoS One. 2012 Oct 25;7(10):e48031. doi: 10.1371/journal.pone.0048031 (PMC3485049; doi:10.1371/journal.pone.0048031)
Supplement: Table S3 — Relationship between C-erbB2 status in breast cancer patients and variants detected in the CD28 gene. 1C-erbB2 information of 479 breast cancer patients was available in the study with 187 (33.10%) positive and 292 (51.68%) negative ones. 2The P values were accessed using Plink and SPSS software under an additive model (AA vs. Aa vs. aa), dominant model (aa+Aa vs. AA), and recessive model (aa vs. aA +AA) respectively. Significant values (P<0.05) are in bold. (DOC) [file pone.0048031.s005.doc]

**Table S3 Relationship between C-erbB2 status in breast cancer patients and variants detected in the CD28 gene**

| Relationship with C-erbB2^1^ status on SNP level | | | | | | |  | Relationship with C-erbB2^1^ status on Haplotype level | | | | |
| --- | --- | --- | --- | --- | --- | --- | --- | --- | --- | --- | --- | --- |
| SNP ID | "a"* | "A"* | Model^2^ | Positive | Negative | *P* value |  | Haplotype | Freq. | Positive, negative ratios | Chi-square | *P* value |
| rs3181097 | A | G | Additive | 46/90/51 | 62/152/78 | 0.6269 |  |  |  |  |  |  |
| rs3181097 | A | G | Allelic | 182/192 | 276/308 | 0.6715 |  | BLOCK1 |  |  |  |  |
| rs3181097 | A | G | Dominant | 136/51 | 214/78 | 0.8927 |  | BLOCK1-AGCTCCC | 0.449 | 168.8 : 205.2, 261.1 : 322.9 | 0.017 | 0.8955 |
| rs3181097 | A | G | Recessive | 46/141 | 62/230 | 0.3898 |  | BLOCK1-GACCTTT | 0.225 | 80.3 : 293.7, 135.0 : 449.0 | 0.356 | 0.5506 |
| rs35593994 | A | G | Additive | 11/75/101 | 22/110/160 | 0.7263 |  | BLOCK1-GGGCCTT | 0.137 | 53.5 : 320.5, 78.1 : 505.9 | 0.171 | 0.6792 |
| rs35593994 | A | G | Allelic | 97/277 | 154/430 | 0.8815 |  | BLOCK1-GGCCTTT | 0.058 | 24.3 : 349.7, 31.5 : 552.5 | 0.494 | 0.4820 |
| rs35593994 | A | G | Dominant | 86/101 | 132/160 | 0.8665 |  | BLOCK1-GGCCCTT | 0.016 | 6.2 : 367.8, 8.9 : 575.1 | 0.030 | 0.8627 |
| rs35593994 | A | G | Recessive | 11/176 | 22/270 | 0.4862 |  | BLCOK1-GGCTCCC | 0.015 | 4.0 : 370.0, 10.2 : 573.8 | 0.726 | 0.3941 |
| rs3181100 | G | C | Additive | 5/57/125 | 10/79/203 | 0.6717 |  | **BLOCK1-GGCTCTC** | 0.013 | 1.0 : 373.0, 11.5 : 572.5 | 5.077 | **0.0242** |
| rs3181100 | G | C | Allelic | 67/307 | 99/485 | 0.7010 |  |  |  |  |  |  |
| rs3181100 | G | C | Dominant | 62/125 | 89/203 | 0.5386 |  | BLOCK 2 |  |  |  |  |
| rs3181100 | G | C | Recessive | 5/182 | 10/282 | 0.6453 |  | **BLOCK2-CA** | 0.934 | 341.0:33.0,554.0:30.0 | 5.043 | **0.0247** |
| rs1181388 | C | T | Additive | 48/95/44 | 65/151/76 | 0.6493 |  | **BLOCK2-GG** | 0.066 | 33.0:341.0,30.0:554.0 | 5.043 | **0.0247** |
| rs1181388 | C | T | Allelic | 191/183 | 281/303 | 0.3725 |  |  |  |  |  |  |
| rs1181388 | C | T | Dominant | 143/44 | 216/76 | 0.5382 |  |  |  |  |  |  |
| rs1181388 | C | T | Recessive | 48/139 | 65/227 | 0.3914 |  |  |  |  |  |  |
| rs10932017 | T | C | Additive | 16/89/82 | 22/135/135 | 0.8445 |  |  |  |  |  |  |
| rs10932017 | T | C | Allelic | 121/253 | 179/405 | 0.5794 |  |  |  |  |  |  |
| rs10932017 | T | C | Dominant | 105/82 | 157/135 | 0.6093 |  |  |  |  |  |  |
| rs10932017 | T | C | Recessive | 16/171 | 22/270 | 0.6864 |  |  |  |  |  |  |
| rs4673259 | C | T | Additive | 44/94/49 | 67/153/72 | 0.8936 |  |  |  |  |  |  |
| rs4673259 | C | T | Allelic | 182/192 | 287/297 | 0.8845 |  |  |  |  |  |  |
| rs4673259 | C | T | Dominant | 138/49 | 220/72 | 0.7041 |  |  |  |  |  |  |
| rs4673259 | C | T | Recessive | 44/143 | 67/225 | 0.8825 |  |  |  |  |  |  |
| rs3769684 | T | C | Additive | 46/91/50 | 64/152/76 | 0.7272 |  |  |  |  |  |  |
| rs3769684 | T | C | Allelic | 183/191 | 280/304 | 0.7659 |  |  |  |  |  |  |
| rs3769684 | T | C | Dominant | 137/50 | 216/76 | 0.8632 |  |  |  |  |  |  |
| rs3769684 | T | C | Recessive | 46/141 | 64/228 | 0.4961 |  |  |  |  |  |  |
| rs3116487 | G | C | Allelic | 33/341 | 30/554 | **0.0247** |  |  |  |  |  |  |
| rs3116487 | G | C | Dominant | 33/154 | 30/262 | **0.0198** |  |  |  |  |  |  |
| rs3116494 | G | A | Allelic | 33/341 | 30/554 | **0.0247** |  |  |  |  |  |  |
| rs3116494 | G | A | Dominant | 33/154 | 30/262 | **0.0198** |  |  |  |  |  |  |
| rs3116496 | C | T | Additive | 3/33/151 | 2/53/237 | 0.6437 |  |  |  |  |  |  |
| rs3116496 | C | T | Allelic | 39/335 | 57/527 | 0.7371 |  |  |  |  |  |  |
| rs3116496 | C | T | Dominant | 36/151 | 55/237 | 0.9099 |  |  |  |  |  |  |
| rs3116496 | C | T | Recessive | 3/184 | 2/290 | 0.3833 |  |  |  |  |  |  |
| rs12693993 | A | G | Additive | 6/51/130 | 6/75/211 | 0.6586 |  |  |  |  |  |  |
| rs12693993 | A | G | Allelic | 63/311 | 87/497 | 0.4184 |  |  |  |  |  |  |
| rs12693993 | A | G | Dominant | 57/130 | 81/211 | 0.5181 |  |  |  |  |  |  |
| rs12693993 | A | G | Recessive | 6/181 | 6/286 | 0.4306 |  |  |  |  |  |  |
| rs3769686 | G | A | Allelic | 5/369 | 12/572 | 0.4116 |  |  |  |  |  |  |
| rs3769686 | G | A | Dominant | 5/182 | 12/280 | 0.4074 |  |  |  |  |  |  |

^1^ C-erbB2 information of 479 breast cancer patients was available in the study with 187 (33.10%) positive and 292 (51.68%) negative ones.

^2^The *P* values were accessed using Plink and SPSS software under an additive model (AA vs. Aa vs. aa), dominant model (aa+Aa vs. AA), and recessive model (aa vs. aA +AA) respectively. Significant values (*P* <0.05) are in bold.

*Minor allele ‘a’ and the major ‘A’ are shown in the table. ‘AA’, ‘Aa’, ‘aa’ represent a given variant for each SNP genotyped.
