# Supplementary material for: Investigation of CD28 Gene Polymorphisms in Patients with Sporadic Breast Cancer in a Chinese Han Population in Northeast China
Source: PLoS One. 2012 Oct 25;7(10):e48031. doi: 10.1371/journal.pone.0048031 (PMC3485049; doi:10.1371/journal.pone.0048031)
Supplement: Table S4 — Relationship between P53 status in breast cancer patients and variants detected in the CD28 gene. 1P53 information of 467 breast cancer patients was available in the study with 146 (25.84%) positive and 321 (56.81%) negative ones. 2The P values were accessed using Plink and SPSS software under an additive model (AA vs. Aa vs. aa), dominant model (aa+Aa vs. AA), and recessive model (aa vs. aA+AA) respectively. Significant values (P<0.05) are in bold. (DOC) [file pone.0048031.s006.doc]

**Table S4 Relationship between P53 status in breast cancer patients and variants detected in the CD28 gene**

| Relationship with P53^1^ status on SNP level | | | | | | |  | Relationship with P53^1^ status on Haplotype level | | | | |
| --- | --- | --- | --- | --- | --- | --- | --- | --- | --- | --- | --- | --- |
| SNP ID | "a"* | "A"* | Model^2^ | Positive | Negative | *P* value |  | Haplotype | Freq. | Positive, negative ratios | Chi-square | *P* value |
| rs3181097 | A | G | Additive | 31/73/42 | 75/161/85 | 0.8210 |  |  |  |  |  |  |
| rs3181097 | A | G | Allelic | 135/157 | 311/331 | 0.5309 |  | BLOCK1-AGCTCCC | 0.446 | 121.2 : 170.8, 295.1 : 346.9 | 1.609 | 0.2046 |
| rs3181097 | A | G | Dominant | 104/42 | 236/85 | 0.6066 |  | BLOCK1-GACCTTT | 0.226 | 66.5 : 225.5, 144.7 : 497.3 | 0.007 | 0.9325 |
| rs3181097 | A | G | Recessive | 31/115 | 75/246 | 0.6102 |  | BLOCK1-GGGCCTT | 0.139 | 45.0 : 247.0, 84.6 : 557.4 | 0.833 | 0.3613 |
| rs35593994 | A | G | Additive | 12/56/78 | 20/126/175 | 0.7327 |  | BLOCK1-GGCCTTT | 0.059 | 14.9 : 277.1, 39.7 : 602.3 | 0.422 | 0.5159 |
| rs35593994 | A | G | Allelic | 80/212 | 166/476 | 0.6202 |  | BLOCK1-GGCCCTT | 0.016 | 4.1 : 287.9, 11.1 : 630.9 | 0.138 | 0.7100 |
| rs35593994 | A | G | Dominant | 68/78 | 146/175 | 0.8261 |  | BLCOK1-GGCTCCC | 0.015 | 5.0 : 287.0, 9.0 : 633.0 | 0.133 | 0.7157 |
| rs35593994 | A | G | Recessive | 12/134 | 20/301 | 0.4304 |  | BLOCK1-GGCTCTC | 0.011 | 5.0 : 287.0, 9.0 : 633.0 | 1.770 | 0.1834 |
| rs3181100 | G | C | Additive | 7/45/94 | 8/87/226 | 0.2621 |  |  |  |  |  |  |
| rs3181100 | G | C | Allelic | 59/233 | 103/539 | 0.1194 |  | BLOCK 2 |  |  |  |  |
| rs3181100 | G | C | Dominant | 52/94 | 95/226 | 0.1940 |  | BLOCK2-CA | 0.935 | 272.0 : 20.0, 601.0 : 41.0 | 0.007 | 0.7906 |
| rs3181100 | G | C | Recessive | 7/139 | 8/313 | 0.1908 |  | BLOCK2-GG | 0.065 | 20.0 : 272.0, 41.0 : 601.0 | 0.007 | 0.7906 |
| rs1181388 | C | T | Additive | 36/74/36 | 76/166/79 | 0.9699 |  |  |  |  |  |  |
| rs1181388 | C | T | Allelic | 146/146 | 318/324 | 0.8947 |  |  |  |  |  |  |
| rs1181388 | C | T | Dominant | 110/36 | 242/79 | 0.9913 |  |  |  |  |  |  |
| rs1181388 | C | T | Recessive | 36/110 | 76/245 | 0.8179 |  |  |  |  |  |  |
| rs10932017 | T | C | Additive | 11/67/68 | 25/154/142 | 0.8947 |  |  |  |  |  |  |
| rs10932017 | T | C | Allelic | 89/203 | 204/438 | 0.6923 |  |  |  |  |  |  |
| rs10932017 | T | C | Dominant | 78/68 | 179/142 | 0.6377 |  |  |  |  |  |  |
| rs10932017 | T | C | Recessive | 11/135 | 25/296 | 0.9240 |  |  |  |  |  |  |
| rs4673259 | C | T | Additive | 35/72/39 | 74/166/81 | 0.8891 |  |  |  |  |  |  |
| rs4673259 | C | T | Allelic | 142/150 | 314/328 | 0.9369 |  |  |  |  |  |  |
| rs4673259 | C | T | Dominant | 107/39 | 240/81 | 0.7346 |  |  |  |  |  |  |
| rs4673259 | C | T | Recessive | 35/111 | 74/247 | 0.8276 |  |  |  |  |  |  |
| rs3769684 | T | C | Additive | 40/70/36 | 69/166/86 | 0.3763 |  |  |  |  |  |  |
| rs3769684 | T | C | Allelic | 150/142 | 304/338 | 0.2548 |  |  |  |  |  |  |
| rs3769684 | T | C | Dominant | 110/36 | 235/86 | 0.6266 |  |  |  |  |  |  |
| rs3769684 | T | C | Recessive | 40/106 | 69/252 | 0.1622 |  |  |  |  |  |  |
| rs3116487 | G | C | Allelic | 20/272 | 41/601 | 0.7906 |  |  |  |  |  |  |
| rs3116487 | G | C | Dominant | 20/126 | 41/280 | 0.7831 |  |  |  |  |  |  |
| rs3116494 | G | A | Allelic | 20/272 | 41/601 | 0.7906 |  |  |  |  |  |  |
| rs3116494 | G | A | Dominant | 20/126 | 41/280 | 0.7831 |  |  |  |  |  |  |
| rs3116496 | C | T | Additive | 2/22/122 | 3/62/256 | 0.5118 |  |  |  |  |  |  |
| rs3116496 | C | T | Allelic | 26/266 | 68/574 | 0.4267 |  |  |  |  |  |  |
| rs3116496 | C | T | Dominant | 24/122 | 65/256 | 0.3310 |  |  |  |  |  |  |
| rs3116496 | C | T | Recessive | 2/144 | 3/318 | 0.6501 |  |  |  |  |  |  |
| rs12693993 | A | G | Additive | 5/43/98 | 7/80/234 | 0.3952 |  |  |  |  |  |  |
| rs12693993 | A | G | Allelic | 53/239 | 94/548 | 0.1722 |  |  |  |  |  |  |
| rs12693993 | A | G | Dominant | 48/98 | 87/234 | 0.2020 |  |  |  |  |  |  |
| rs12693993 | A | G | Recessive | 5/141 | 7/314 | 0.4309 |  |  |  |  |  |  |
| rs3769686 | G | A | Allelic | 3/289 | 14/628 | 0.2216 |  |  |  |  |  |  |
| rs3769686 | G | A | Dominant | 3/143 | 14/307 | 0.2173 |  |  |  |  |  |  |

^1^P53 information of 467 breast cancer patients was available in the study with 146 (25.84%) positive and 321 (56.81%) negative ones.

^2^The *P* values were accessed using Plink and SPSS software under an additive model (AA vs. Aa vs. aa), dominant model (aa+Aa vs. AA), and recessive model (aa vs. aA+AA) respectively. Significant values (*P* <0.05) are in bold.

*Minor allele ‘a’ and the major ‘A’ are shown in the table. ‘AA’, ‘Aa’, ‘aa’ represent a given variant for each SNP genotyped.
