# Supplementary material for: Investigation of CD28 Gene Polymorphisms in Patients with Sporadic Breast Cancer in a Chinese Han Population in Northeast China
Source: PLoS One. 2012 Oct 25;7(10):e48031. doi: 10.1371/journal.pone.0048031 (PMC3485049; doi:10.1371/journal.pone.0048031)
Supplement: Table S5 — Relationship between lymph node involvement status in breast cancer patients and variants detected in the CD28 gene. 1LN, lymph node. 2LN involvement information of 543 breast cancer patients was available in the study with 235 (41.59%) positive and 310 (54.87%) negative ones. 3The P values were accessed using Plink and SPSS software under an additive model (AA vs. Aa vs. aa), dominant model (aa+Aa vs. AA), and recessive model (aa vs. aA+AA) respectively. Significant values (P<0.05) are in bold. (DOC) [file pone.0048031.s007.doc]

**Table S5 Relationship between lymph node involvement status in breast cancer patients and variants detected in the CD28 gene**

| Relationship with LN^1^ involvement^2^ status on SNP level | | | | | | |  | Relationship with LN involvement^2^ status on Haplotype level | | | | |
| --- | --- | --- | --- | --- | --- | --- | --- | --- | --- | --- | --- | --- |
| SNP ID | "a"* | "A"* | Model^3^ | Positive | Negative | *P* value |  | Haplotype | Freq. | Positive, negative ratios | Chi-square | *P* value |
| rs3181097 | A | G | Additive | 51/123/61 | 77/150/83 | 0.6033 |  |  |  |  |  |  |
| rs3181097 | A | G | Allelic | 225/245 | 304/316 | 0.7043 |  | BLOCK1 |  |  |  |  |
| rs3181097 | A | G | Dominant | 174/61 | 227/83 | 0.8304 |  | BLOCK1-AGCTCCC | 0.457 | 210.3 : 255.7, 284.2 : 331.8 | 0.106 | 0.7449 |
| rs3181097 | A | G | Recessive | 51/184 | 77/233 | 0.3923 |  | BLOCK1-GACCTTT | 0.218 | 98.5 : 367.5, 137.6 : 478.4 | 0.223 | 0.6367 |
| rs35593994 | A | G | Additive | 15/82/138 | 21/126/163 | 0.3494 |  | BLOCK1-GGGCCTT | 0.132 | 68.1 : 397.9, 75.1 : 540.9 | 1.351 | 0.2452 |
| rs35593994 | A | G | Allelic | 112/358 | 168/452 | 0.2215 |  | BLOCK1-GGCCTTT | 0.065 | 32.9 : 433.1, 37.1 : 578.9 | 0.474 | 0.4910 |
| rs35593994 | A | G | Dominant | 97/138 | 147/163 | 0.1532 |  | BLOCK1-GGCCCTT | 0.015 | 8.3 : 457.7, 8.0 : 608.0 | 0.420 | 0.5168 |
| rs35593994 | A | G | Recessive | 15/220 | 21/289 | 0.8555 |  | BLOCK1-GGCTCTC | 0.015 | 6.9 : 459.1, 9.2 : 606.8 | 0.001 | 0.9740 |
| rs3181100 | G | C | Additive | 9/68/158 | 8/84/218 | 0.6018 |  | BLCOK1-GGCTCCC | 0.013 | 4.9 : 461.1, 8.9 : 607.1 | 0.328 | 0.5666 |
| rs3181100 | G | C | Allelic | 86/384 | 100/520 | 0.3459 |  |  |  |  |  |  |
| rs3181100 | G | C | Dominant | 77/158 | 92/218 | 0.4401 |  | BLOCK 2 |  |  |  |  |
| rs3181100 | G | C | Recessive | 9/226 | 8/302 | 0.4061 |  | BLOCK2-CA | 0.928 | 433.0 : 37.0, 578.0 : 42.0 | 0.480 | 0.4886 |
| rs1181388 | C | T | Additive | 59/117/59 | 67/160/83 | 0.6263 |  | BLOCK2-GG | 0.072 | 37.0 : 433.0, 42.0 : 578.0 | 0.480 | 0.4886 |
| rs1181388 | C | T | Allelic | 235/235 | 294/326 | 0.3985 |  |  |  |  |  |  |
| rs1181388 | C | T | Dominant | 176/59 | 227/83 | 0.6604 |  |  |  |  |  |  |
| rs1181388 | C | T | Recessive | 59/176 | 67/243 | 0.3380 |  |  |  |  |  |  |
| rs10932017 | T | C | Additive | 18/110/107 | 25/144/141 | 0.9844 |  |  |  |  |  |  |
| rs10932017 | T | C | Allelic | 146/324 | 194/426 | 0.9363 |  |  |  |  |  |  |
| rs10932017 | T | C | Dominant | 128/107 | 169/141 | 0.9911 |  |  |  |  |  |  |
| rs10932017 | T | C | Recessive | 18/217 | 25/285 | 0.8621 |  |  |  |  |  |  |
| rs4673259 | C | T | Additive | 53/118/64 | 78/161/71 | 0.4800 |  |  |  |  |  |  |
| rs4673259 | C | T | Allelic | 224/246 | 317/303 | 0.2566 |  |  |  |  |  |  |
| rs4673259 | C | T | Dominant | 171/64 | 239/71 | 0.2461 |  |  |  |  |  |  |
| rs4673259 | C | T | Recessive | 53/182 | 78/232 | 0.4804 |  |  |  |  |  |  |
| rs3769684 | T | C | Additive | 54/120/61 | 69/154/87 | 0.8605 |  |  |  |  |  |  |
| rs3769684 | T | C | Allelic | 228/242 | 292/328 | 0.6435 |  |  |  |  |  |  |
| rs3769684 | T | C | Dominant | 174/61 | 223/87 | 0.5839 |  |  |  |  |  |  |
| rs3769684 | T | C | Recessive | 54/181 | 69/241 | 0.8420 |  |  |  |  |  |  |
| rs3116487 | G | C | Allelic | 37/433 | 42/578 | 0.4886 |  |  |  |  |  |  |
| rs3116487 | G | C | Dominant | 37/198 | 42/268 | 0.4707 |  |  |  |  |  |  |
| rs3116494 | G | A | Allelic | 37/433 | 42/578 | 0.4886 |  |  |  |  |  |  |
| rs3116494 | G | A | Dominant | 37/198 | 42/268 | 0.4707 |  |  |  |  |  |  |
| rs3116496 | C | T | Additive | 5/44/186 | 1/60/249 | 0.1692 |  |  |  |  |  |  |
| rs3116496 | C | T | Allelic | 54/416 | 62/558 | 0.4297 |  |  |  |  |  |  |
| rs3116496 | C | T | Dominant | 49/186 | 61/249 | 0.7353 |  |  |  |  |  |  |
| rs3116496 | C | T | Recessive | 5/230 | 1/309 | 0.0895 |  |  |  |  |  |  |
| rs12693993 | A | G | Additive | 7/66/162 | 6/76/228 | 0.4359 |  |  |  |  |  |  |
| rs12693993 | A | G | Allelic | 80/390 | 88/532 | 0.2004 |  |  |  |  |  |  |
| rs12693993 | A | G | Dominant | 73/162 | 82/228 | 0.2372 |  |  |  |  |  |  |
| rs12693993 | A | G | Recessive | 7/228 | 6/304 | 0.4293 |  |  |  |  |  |  |
| rs3769686 | G | A | Allelic | 10/460 | 10/610 | 0.5306 |  |  |  |  |  |  |
| rs3769686 | G | A | Dominant | 10/225 | 10/300 | 0.5267 |  |  |  |  |  |  |

^1^LN= lymph node

^2^LN involvement information of 543 breast cancer patients was available in the study with 235 (41.59%) positive and 310 (54.87%) negative ones.

^3^The *P* values were accessed using Plink and SPSS software under an additive model (AA vs. Aa vs. aa), dominant model (aa+Aa vs. AA), and recessive model (aa vs. aA +AA) respectively. Significant values (*P* <0.05) are in bold.

*Minor allele ‘a’ and the major ‘A’ are shown in the table. ‘AA’, ‘Aa’, ‘aa’ represent a given variant for each SNP genotyped.
