# Supplementary material for: Investigation of CD28 Gene Polymorphisms in Patients with Sporadic Breast Cancer in a Chinese Han Population in Northeast China
Source: PLoS One. 2012 Oct 25;7(10):e48031. doi: 10.1371/journal.pone.0048031 (PMC3485049; doi:10.1371/journal.pone.0048031)
Supplement: Table S6 — Association between tumor size and polymorphisms. 1466 tumor size information in detail of breast cancer patients were available. 2I: Tumor with the diameter less than 2 cm (0<tumor size< = 2), number = 187. 3II: Tumor with the diameter of 2 to 5 cm (2<tumor size< = 5), number = 252. 4III: Tumor with the diameter more than 5 (tumor size>5), number = 27. (DOC) [file pone.0048031.s008.doc]

**Table S6 Association between tumor size and polymorphisms**

| SNP ID | Genotype | Tumor size^1^ | | | Global *P* |
| --- | --- | --- | --- | --- | --- |
|  |  | I^2^ | II^3^ | III^4^ |  |
| rs3181097 | AA | 39 | 59 | 6 |  |
|  | AG | 99 | 127 | 14 | 0.9781 |
|  | GG | 49 | 66 | 7 |  |
| rs35593994 | AA | 12 | 14 | 3 |  |
|  | AG | 62 | 110 | 10 | 0.1882 |
|  | GG | 113 | 128 | 14 |  |
| rs3181100 | GG | 9 | 5 | 1 |  |
|  | GC | 54 | 65 | 8 | 0.3702 |
|  | CC | 124 | 182 | 18 |  |
| rs1181388 | CC | 43 | 56 | 7 |  |
|  | CT | 101 | 131 | 14 | 0.9578 |
|  | TT | 43 | 65 | 6 |  |
| rs10932017 | TT | 12 | 20 | 2 |  |
|  | TC | 86 | 127 | 13 | 0.8044 |
|  | CC | 89 | 105 | 12 |  |
| rs4673259 | CC | 39 | 61 | 6 |  |
|  | CT | 104 | 128 | 14 | 0.8894 |
|  | TT | 44 | 63 | 7 |  |
| rs3769684 | TT | 42 | 55 | 7 |  |
|  | TC | 99 | 129 | 13 | 0.9678 |
|  | CC | 46 | 68 | 7 |  |
| rs3116487 | GG | 0 | 0 | 0 |  |
|  | GC | 29 | 35 | 2 | 0.5201 |
|  | CC | 158 | 217 | 25 |  |
| rs3116494 | GG | 0 | 0 | 0 |  |
|  | GA | 29 | 35 | 2 | 0.5201 |
|  | GG | 158 | 217 | 25 |  |
| rs3116496 | CC | 2 | 3 | 0 |  |
|  | CT | 34 | 48 | 5 | 0.9950 |
|  | TT | 151 | 201 | 22 |  |
| rs12693993 | AA | 10 | 3 | 0 |  |
|  | AG | 51 | 63 | 7 | 0.1103 |
|  | GG | 126 | 186 | 20 |  |
| rs3769686 | GG | 0 | 0 | 0 |  |
|  | GA | 6 | 10 | 1 | 0.9225 |
|  | AA | 181 | 242 | 26 |  |

^1^466 tumor size information in detail of breast cancer patients were available.

^2^I: Tumor with the diameter less than 2 cm (0<tumor size<=2), number=187.

^3^II: Tumor with the diameter of 2 to 5 cm (2<tumor size<=5), number=252.

^4^III: Tumor with the diameter more than 5 (tumor size>5), number=27.
