# Supplementary material for: Investigation of CD28 Gene Polymorphisms in Patients with Sporadic Breast Cancer in a Chinese Han Population in Northeast China
Source: PLoS One. 2012 Oct 25;7(10):e48031. doi: 10.1371/journal.pone.0048031 (PMC3485049; doi:10.1371/journal.pone.0048031)
Supplement: Table S7 — Genotyping information for 14 selected SNPs. 1NEB Cutter, names of restriction enzymes purchased from the NEB biolabs company. (DOC) [file pone.0048031.s009.doc]

**Table S7 Genotyping information for 14 selected SNPs**

| SNP ID | Forward and reverse primers (5’-3’) | NEB cutter^1^ | Annealing  temperature | product  length |
| --- | --- | --- | --- | --- |
| rs1879877 | F 5’-GGAGGTTCCCTAAGCAAAG-3’ | EarI | 56.0°C | 231bp |
|  | R 5’-CCGTGCTGGTGGAATAAC-3’ |  |  |  |
| rs3181097 | F 5’-CCCCCAACTAGAGTAATTGCACA | BstEII | 55.0°C | 342bp |
|  | R 5’-AAAGGGAAAGTCAAAACAACATT  CTATGTTCTTAGTGGGACAGTAAGGTAA-3’ |  |  |  |
| rs35593994 | F 5’-TCCTTCTTTTCTTTTCTTTTCTTTTC-3’ | HinfI | 58.6°C | 550bp |
|  | R 5’-CTGCAGCATTTCACACAGGA-3’ |  |  |  |
| rs3181100 | F 5’-ATTAGTTTCTCTAGCTATTAG  TTGATAGTGTCCC-3’ | BlpI | 55.0°C | 362bp |
|  | R 5’-AGGTTTGGAGAGTTTAAATAATAC  CTAAGCC-3’ |  |  |  |
| rs2140148 | F 5’-AGTTACGAGAACTGTGTGC-3’ | BspCNI | 55.0°C | 327bp |
|  | R 5’-TCCTTAACTGTAAAGTGGG-3’ |  |  |  |
| rs1181388 | F 5’-GTAGTTCTTGATCCTATCCAG-3’ | MnlI | 56.0°C | 316bp |
|  | R 5’-TGACTGACTGAATGGCTC-3’ |  |  |  |
| rs10932017 | F 5’-GTTCTTACCAATGCTGTGTTG-3’ | BlpI | 54.0°C | 270bp |
|  | R 5’-ATTCCCATAACTCCAGCTTC-3’ |  |  |  |
| rs4673259 | F 5’-AATTGCATGACTTGAGCTTC-3’ | PshAI | 58.6°C | 301bp |
|  | R 5’-CCTCTCTCTCCCAGAAATTC-3’ |  |  |  |
| rs3769684 | F 5’-ATCTAAGCATTAACTGAGCTGG-3’ | NcoI | 55.0°C | 293bp |
|  | R 5’-TTTCCCTCACAGCTCCTG-3’ |  |  |  |
| rs3116487 | F 5’-AGGGAGGAAAGTAAACATGAGC-3’ | MwoI | 55.0°C | 278bp |
|  | R 5’-GGAGATGAGGAGGGTAAGAGTG-3’ |  |  |  |
| rs3116494 | F 5’-ATTCCAGTGGATCATGGC-3’ | MslI | 56.0°C | 153bp |
|  | R 5’-TTACAGAGGAAGATTATGGAGC-3’ |  |  |  |
| rs3116496 | F5’-TCAGGGAAACACCTTTGTCCAAGTC-3’ | BtsI | 55.0°C | 188bp |
|  | R 5’-TGTGTTCAGATTTCATGTGCATTGG-3’ |  |  |  |
| rs12693993 | F 5’-AATTGATAACCTCCAGAGAG-3’ | NdeI | 60.0°C | 260bp |
|  | R 5’-GGTAAGCTTTGGCAATAG-3’ |  |  |  |
| rs3769686 | F 5’-ATCCTCTGGTGTTTGCTG-3’ | BsrI | 56.8°C | 293bp |
|  | R 5’-TTCAATAATGAGCAGTGTCC-3’ |  |  |  |

^1^NEB Cutter, names of restriction enzymes purchased from the NEB biolabs company.
